# A New Anæsthetic

**Published:** 1861-06

**Authors:** 


A New Anæsthetic.—A writer in the *Lancet* states that the vapor of turpentine induces anæsthesia. The first case in which he employed it was neuralgia of the supra-orbital nerve; the turpentine was sprinkled on a handkerchief, and inhaled in the same manner as chloroform. After a few inhalations a gentle sleep ensued, from which the patient awoke free from headache, or other unpleasant symptoms. He has since used it in slight operations, cramps, nervous irritation, etc., and found that it induced anæsthetic sleep.—*American Medical Times.*

